# Declining Levels and Bioavailability of IGF-I in Cardiovascular Aging Associate With QT Prolongation–Results From the 1946 British Birth Cohort

**DOI:** 10.3389/fcvm.2022.863988

**Published:** 2022-04-22

**Authors:** Christos Charalambous, James C. Moon, Jeff M. P. Holly, Nishi Chaturvedi, Alun D. Hughes, Gabriella Captur

**Affiliations:** ^1^UCL MRC Unit for Lifelong Health and Ageing, University College London, London, United Kingdom; ^2^UCL Institute of Cardiovascular Science, University College London, London, United Kingdom; ^3^Cardiac MRI Unit, Barts Heart Centre, London, United Kingdom; ^4^National Institute for Health Research (NIHR) Bristol Nutrition Biomedical Research Unit, Level 3, University Hospitals Bristol Education and Research Centre, Bristol, United Kingdom; ^5^Faculty of Health Sciences, School of Translational Health Sciences, Bristol Medical School, Southmead Hospital, University of Bristol, Bristol, United Kingdom; ^6^Cardiology Department, Centre for Inherited Heart Muscle Conditions, The Royal Free Hospital, London, United Kingdom

**Keywords:** QTc interval prolongation, cardiac repolarization, IGF-I (insulin-like growth factor-I), IGFBP-3, IGF-I/IGFBP-3 molar ratio, IGF-II

## Abstract

**Background:**

As people age, circulating levels of insulin-like growth factors (IGFs) and IGF binding protein 3 (IGFBP-3) decline. In rat cardiomyocytes, IGF-I has been shown to regulate sarcolemmal potassium channel activity and late sodium current thus impacting cardiac repolarization and the heart rate-corrected QT (QTc). However, the relationship between IGFs and IGFBP-3 with the QTc interval in humans, is unknown.

**Objectives:**

To examine the association of IGFs and IGFBP-3 with QTc interval in an older age population-based cohort.

**Methods:**

Participants were from the 1946 Medical Research Council (MRC) National Survey of Health and Development (NSHD) British birth cohort. Biomarkers from blood samples at age 53 and 60–64 years (y, exposures) included IGF-I/II, IGFBP-3, IGF-I/IGFBP-3 ratio and the change (Δ) in marker levels between the 60–64 and 53y sampled timepoints. QTc (outcome) was recorded from electrocardiograms at the 60–64y timepoint. Generalized linear multivariable models with adjustments for relevant demographic and clinical factors, were used for complete-cases and repeated after multiple imputation.

**Results:**

One thousand four hundred forty-eight participants were included (48.3% men; QTc mean 414 ms interquartile range 26 ms). Univariate analysis revealed an association between low IGF-I and IGF-I/IGFBP-3 ratio at 60–64y with QTc prolongation [respectively: β −0.30 ms/nmol/L, (95% confidence intervals −0.44, −0.17), *p* < 0.001; β−28.9 ms/unit (-41.93, −15.50), *p* < 0.001], but not with IGF-II or IGFBP-3. No association with QTc was found for IGF biomarkers sampled at 53y, however both ΔIGF-I and ΔIGF-I/IGFBP-3 ratio were negatively associated with QTc [β −0.04 ms/nmol/L (−0.08, −0.008), *p* = 0.019; β −2.44 ms/unit (-4.17, −0.67), *p* = 0.007] while ΔIGF-II and ΔIGFBP-3 showed no association. In fully adjusted complete case and imputed models (reporting latter) low IGF-I and IGF-I/IGFBP-3 ratio at 60–64y [β −0.21 ms/nmol/L (−0.39, −0.04), *p* = 0.017; β −20.14 ms/unit (−36.28, −3.99), *p* = 0.015], steeper decline in ΔIGF-I [β −0.05 ms/nmol/L/10 years (−0.10, −0.002), *p* = 0.042] and shallower rise in ΔIGF-I/IGFBP-3 ratio over a decade [β −2.16 ms/unit/10 years (−4.23, −0.09), *p* = 0.041], were all independently associated with QTc prolongation. Independent associations with QTc were also confirmed for other previously known covariates: female sex [β 9.65 ms (6.65, 12.65), *p* < 0.001], increased left ventricular mass [β 0.04 ms/g (0.02, 0.06), *p* < 0.001] and blood potassium levels [β −5.70 ms/mmol/L (−10.23, −1.18) *p* = 0.014].

**Conclusion:**

Over a decade, in an older age population-based cohort, declining levels and bioavailability of IGF-I associate with prolongation of the QTc interval. As QTc prolongation associates with increased risk for sudden death even in apparently healthy people, further research into the antiarrhythmic effects of IGF-I on cardiomyocytes is warranted.

## Introduction

The QT interval on a 12-lead echocardiogram (ECG) represents the time taken by ventricular cardiomyocytes to depolarize and repolarize. A prolonged QT interval, and especially the T-wave onset to T-peak (1), is thought to result from alterations in sympathetic and parasympathetic activity as well as several other risk factors. Since QTc is a measure of ventricular depolarization and repolarization, having a longer than normal QTc interval risks inducing early afterdepolarizations, and possibly also re-entrant excitation and, torsade de pointes (2–4) ultimately leading to ventricular arrhythmias and ventricular fibrillation (5). Prolongation of the heart rate corrected QT (QTc) is a well-established risk factor for increased cardiovascular mortality (5, 6), all-cause mortality and morbidity (7), even in apparently healthy people (8). Although many of the factors associated with QTc prolongation have been identified, including female sex (9, 10), hypokalemia (11), left ventricular hypertrophy (12), hypertension (13), drug side effects (2) and genetics (14, 15), there are still several unknowns. There is now a need to incorporate metabolic biomarkers in our research to understand the pathophysiology of QTc prolongation.

IGF-I regulates somatic growth, reaching its highest levels during teenage years, with levels decreasing with age (16, 17) and it is involved in cell proliferation, protein synthesis, nutrient homeostasis and nervous system, liver, kidney and cardiac development (18, 19). The decline in IGF-I seems to be greater with higher fat mass (16). IGF-I is also influenced by other hormones, age, sex, diet and nutrition. Previous studies in older age cohorts showed that reduced levels of IGF-I increase the risk of ischemic heart disease and cardiovascular mortality (18, 20). Animal work has shown that insulin like growth factor-I (IGF-I) may influence cardiac repolarization, via the phosphatidyl inositol-3 kinase/protein kinase B (PI3-K/Akt) pathway in cardiomyocytes (21). The PI3-K pathway directly regulates most of the heart's ion channels, including the rapid delayed rectifier potassium channel that specifically influences cardiac repolarization (22) and in some animal models, consequently the QTc duration (23). PI3-K was shown to affect many of the channels involved in the action potential duration, late sodium current, calcium current and slow delayed rectifier potassium channels, through its downstream signaling (22). Yet, little is known about the relationship between IGF-I and QTc duration in humans, particularly in older persons, in whom IGF-I levels are known to decline (24). The insulin growth factor-family (IGFs), including IGF-I and IGF-II, has a wide range of physiological functions including the regulation of cellular proliferation, apoptosis, protein synthesis and metabolism (25). IGF-I in the circulation is bound to insulin like growth factor binding protein-3 (IGFBP-3) and therefore the molar ratio between IGF-I and IGFBP-3 indicates IGF-I bioavailability (26).

We sought to investigate the association between circulating blood levels of IGFs and IGFBP-3 with cardiac repolarization represented by the QTc interval in older age participants of a population-based longitudinal cohort.

## Methods

### Study Population

**Figure 1:**
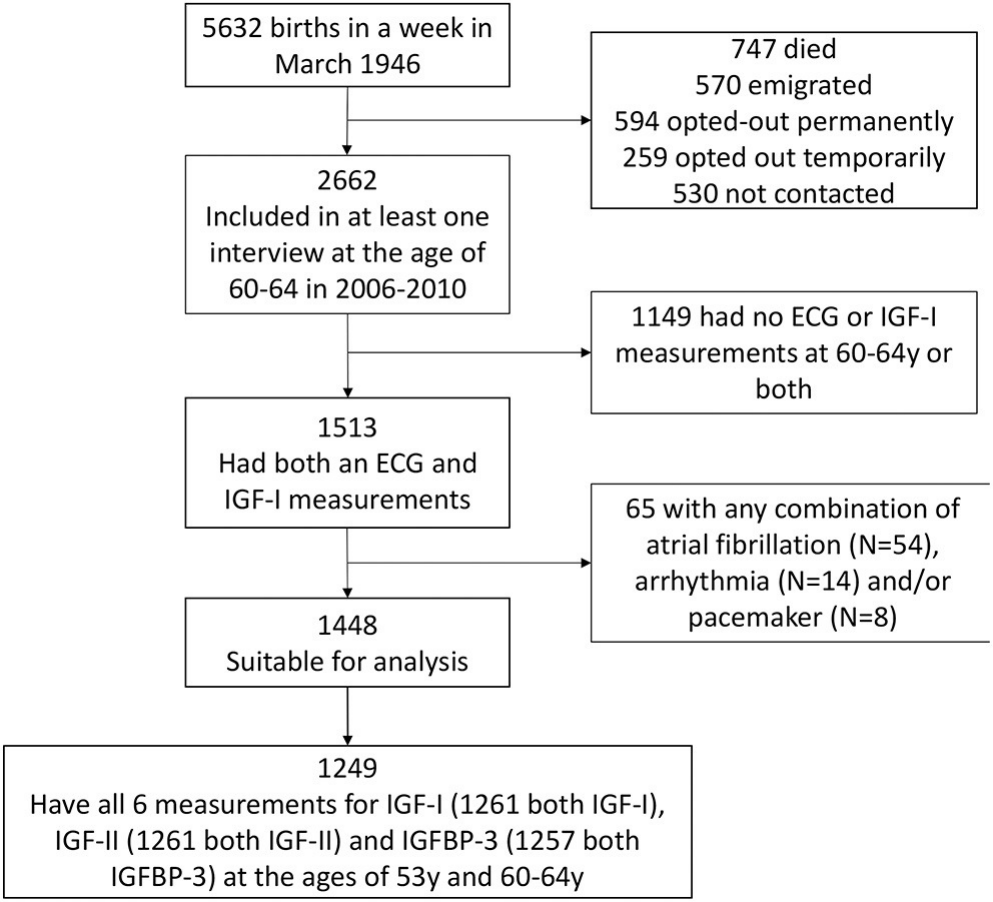
Consort diagram of the recruitment process. The Medical Research Council National Survey of Health and Development (NSHD) consists of 5,362 individuals recruited in 1 week in March 1946 in Britain. The exposures of interest here were metabolic markers at 60–64 years while the outcome was QTc interval derived from resting 12-lead electrocardiography (ECG) during the same clinic visit. Both pieces of data were available for 1,513 out of the 5,362 participants. The number of participants involved int the study is presented in the figure below. ECG, electrocardiography; IGF-I, insulin-like growth factor 1; IGF-BP3, insulin-like growth factor binding protein 3.

Participants were from the Medical Research Council (MRC) National Survey of Health and Development (NSHD), a birth cohort study comprised of 5,362 individuals born in 1 week in 1946 in Britain. The cohort has been in continuous follow up since birth, with 24 data collection cycles (27). Briefly, the cohort has been evaluated multi-dimensionally: anthropometrically, socio-economically (manual and non-manual), and in terms of life-style choices (e.g., smoking) and health function (e.g., mental health, cardiovascular and respiratory function) (27). A consort diagram summarizing the recruitment process for the current study is presented in Figure 1. Previous studies in the NSHD cohort have shown that use of SEP at age 53 as a surrogate for SEP at age 60–64 years is both justifiable and sound, first, because the majority of participants were retired by the age of 60 implying no significant SEP shifts between time-points, and second, because it provided a means to backfill the otherwise high SEP missingness in the cohort at age 60–64 (28, 29).

### Ethical Approval

The 2006–2010 NSHD data collection sweep included an in-depth cardiovascular assessment and was granted ethical approval from the Greater Manchester Local Research Ethics Committee and the Scotland Research Ethics Committee (27) and written informed consent was given by all study participants. All procedures performed were in accordance with the ethical standards of our institutional and/or national research ethics committees and conformed to the 1964 Helsinki declaration and its later amendments or comparable ethical standards.

### Outcome: QTc Interval on ECG at 60–64years

Between 2006 and 2010 when study members were 60–64 years (y), British-based NSHD participants who had not been lost to follow-up or withdrawn, were invited to attend a clinic-based assessment that included a 12-lead resting surface ECG for measurement of QT and R-R intervals using validated computerized algorithms (27). QTc was calculated using Hodges' formula (30):


$$\begin{array}{l} QTc=QT+1.75\left(HR-60\;\right). \end{array}$$

Those with severe conduction system disease requiring a permanent pacemaker and those with any type of a cardiac implantable electronic device (*n*=8), atrial fibrillation (*n*=54) or taking anti-arrhythmic medications (*n*=14) at this period were excluded.

### IGF-I, IGF-II and IGFBP-3

Blood samples were collected at age 53y (non-fasting), and age 60–64y (fasting), stored at −80°C and assayed together. IGF-I and IGF-II and IGFBP-3 concentrations were obtained by radioimmunoassay using standard protocols in the same laboratory, as previously described (31). The intra- and inter-assay coefficients of variation for IGF-I, IGF-II, and IGFBP-3 have been previously reported (16, 32). IGF-I, IGF-II, and IGFBP-3 values were converted from ng/mL to standard SI units (nmol/L) (33, 34) considering: 1 ng/ml IGF-I= 0 .130 nmol/L IGF-I; 1 ng/ml IGF-II = 0.134 nmol/L IGF-II; and 1 ng/ml IGFBP-3 = 0.036 nmol/L IGFBP-3. IGF-I/IGFBP-3 values were expressed as molar ratios to indicate IGF-I bioavailability. In order to standardize individual biomarkers' deviation (Delta, Δ) over the ~10-year period, i.e., from their respective initial 53y timepoint concentrations, the serum concentration at the 60–64y timepoint was divided by its 53y concentration, as used in previous studies (35), then multiplied by the year difference between sampled timepoints divided by the maximum possible year difference, and finally multiplied by 100 to obtain a Δ marker score (Equation 1).


(1) $$\begin{array}{l} \Delta \;Marker\;score=\frac{Marker\;concentration\;at\;60-64y}{Marker\;at\;concentration\;at\;53y}\;X\;\\ \;\frac{Year\;difference\;between\;measurements}{\mathrm{Max}possible\;years\;(i.e.\;11)\;}\;X\;100 \end{array}$$

### Covariates

Covariates were selected a priori, based on previous studies and added into our models successively, after centering on age, to help with the interpretation of coefficients. Model 1 adjusted for sex; Model 2 included additional adjustments for socioeconomic position (SEP); Model 3 added clinical covariates known to be associated with QTc; and Model 4 added cardiac covariates known to be associated with QTc. The same models were used for all the IGF/IGFBP-3 biomarkers, at both time points, including ΔIGFs.

The sex of participants was recorded as male or female. Participants' SEP was evaluated using occupational data from 1989, when they were of active working age, according to the UK Office of Population Censuses and Surveys Registrar General's social class classification and dichotomized into manual or non-manual. Participants' weight and height were used to compute body mass index (BMI). Information about medication usage relevant to the QTc, including antibiotics, antihypertensives, antipsychotics and tricyclic antidepressants (88) was collected through survey instruments and self-reporting along with other relevant clinical information i.e., history of diabetes, heart disease (capturing ischemic heart disease, myocardial infarction, heart failure, heart rhythm abnormality, congenital heart disease, rheumatic heart disease, and other cardiovascular diseases), hypertension, alcohol intake (g per week), physical activity (counting any case of self-reported physical activity carried out over the preceding 4 weeks and 12 months), and smoking into never, ex- or current as previously described (36–38). Additional blood investigations carried out on samples at 60–64y included glucose, glycated hemoglobin A1c, total cholesterol, high- and low-density lipoprotein cholesterol, triglycerides and electrolytes. Assay details have been reported previously (39, 40). At the same clinic visit, two-dimensional transthoracic echocardiography was performed to measure left ventricular ejection fraction and mass (41).

### Statistical Analysis

Statistical analysis was performed in R Studio version 4.0.2 (RStudio Team 2020). Distribution of data was assessed visually using Q-Q plots, histograms and the Shapiro-Wilk test. Continuous sample variables are expressed as mean ± 1 standard deviation (SD) or median (interquartile range) as appropriate; categorical sample variables, as counts and percent. Paired biomarker differences across the decade were investigated by the paired Wilcoxon rank sum test.

As a result of the skewed distribution of continuous QT parameters, generalized linear models (glm) with a gamma distribution and log link were used to investigate the association of IGFs with QTc interval. Model assumptions were verified with regression diagnostics. Multi-collinearity between final model variables was excluded by demonstrating variance inflation factors <3. To determine whether the associations of IGFs and IGFBP-3 with QTc differed by sex or by heart disease, interaction terms for sex and heart disease were tested at the 10% significance level for all exposures and no interactions were found to justify stratification by either sex or heart disease. To account for data missingness, we used multiple imputation to generate missing covariates and re-ran the multivariable models. The predictive mean matching multiple imputation model using chained equations (42) included all the exposures, covariates and outcomes from the fully adjusted multivariable models, and generated 5 sets of covariates. Regression coefficient estimates, and their associated variance metrics were calculated for each dataset and combined using the Rubin's rule. Strength of evidence for an association was assessed on the basis of the size of the regression coefficients, their confidence interval (CI) and the *p*-value after controlling the false discovery rate at 5%. All tests were 2 sided; *p* < 0.05 was considered statistically significant and no adjustment was made for multiple testing.

We ran sensitivity analyses on the imputed models in which we re-analyzed the association between IGFs and IGFBP-3 with QTc after removing participants with known cardiovascular disease, and in which we additionally adjust for heart rate to avoid any remaining confounding.

## Results

### Participant Characteristics

Of the 5,362 originally enrolled into NSHD, 747 were deceased, 570 had emigrated, 853 had withdrawn and 530 were not contactable, leaving 2,662 that were successfully interviewed between 2006 and 2010. Of these 1,513 had contemporaneous ECG for QTc (outcome) and blood tests for the necessary IGFs/IGFBP-3 biomarkers (exposures). A final sample of 1,448 suitable for analysis remained after excluding participants with a device, atrial fibrillation or on anti-arrhythmics. Of these 1,261, 1,261 and 1,257 participants had repeat measures (at 53y and 60–64y) available for IGF-I, IGF-II and IGFBP-3, respectively. Clinicodemographic characteristics of study participants are presented in Table 1. The population mean QTc was 414 ms (IQR 402–428 ms), with 48.3% being male. Sixty-three male participants (9%) had a QTc prolongation, by definition, (>440 ms) compared to 42 female participants (5.6%; QTc >450 ms) (43), acknowledging the fact that QTc prolongation within the normal range can still be associated with cardiac arrhythmia. Participants with longer QTc intervals had higher BMI, lower resting heart rate, blood potassium, and circulating levels of IGF-I. Missing data for each covariate per exposure-outcome pair are presented in Supplementary Tables 1, 2.

**Table 1:** Participant characteristics across the quartiles of QTc.

	**All participants *N* = 1,448**	**Quartiles of QTc**			
		**Quartile 1**	**Quartile 2**	**Quartile 3**	**Quartile 4**
		**(345–402 ms)** ***n* = 376**	**(402–414 ms)** ***n* = 364**	**(414–428 ms)** ***n* = 355**	**(428–529 ms)** ***n* = 353**
**QTc (ms)**	414 (26)	394 (11)	408 (5)	421 (6)	439 (14)
**Demographics**					
Men, *n* (%)	700 (48.3)	217 (57.7)	188 (51.6)	162 (45.6)	133 (37.7)
SEP 1989^a^					
Manual, *n* (%)	378 (26.1)	90 (18.6)	90 (24.7)	94 (26.5)	104 (29.5)
Non-manual, *n* (%)	990 (68.4)	268 (71.3)	255 (70.1)	241 (67.9)	226 (64.0)
**Anthropometric**					
BMI (kg/m^2^)	26.9 ± 5.8	26.4 (5.0)	26.8 (6.1)	26.8 (5.8)	27.5 (6.2)
**ECG**					
SBP (mmHg)	136 (22)	136 (24)	133 (23)	133 (22)	137 (22)
DBP (mmHg)	78 (12)	78 (13)	78 (12)	78 (14)	77 (13)
MAP (mmHg)	96 (15)	97 (15)	97 (14)	95 (16)	96 (15)
Heart rate (ECG)	65 (13)	66 (12)	65 (12)	64 (13)	63 (16)
**Echocardiography**					
LV mass (g)	205.6 (94.7)	200.5 (89.2)	207.0 (81.3)	211.3 (107.4)	203.9 (97.4)
LV EF (%)	65.13 (9.42)	65.22 (9.84)	64.76 (9.28)	65.56 (9.12)	64.88 (9.14)
**Blood markers**					
IGF-I at 53y (nmol/L)	25.2 (10.2)	25.2 (9.7)	25.2 (9.8)	25.2 (10.8)	25.0 (10.4)
IGF-II at 53y (nmol/L)	99.3 (42.6)	99.1 (38.8)	97.3 (42.3)	97.5 (46.4)	102.1 (43.0)
IGFBP-3 at 53y (nmol/L)	171.5 (47.8)	171.2 (48.8)	173.5 (49.0)	172.2 (48.2)	170.0 (45.2)
IGF-I/IGFBP-3 molar ratio at 53y	0.148 (0.065)	0.147 (0.068)	0.151 (0.061)	0.145 (0.064)	0.148 (0.065)
IGF-I at 60–64y (nmol/L)	22.1 (10.0)	22.9 (10.1)	22.8 (10.8)	21.8 (10.6)	20.7 (8.9)
IGF-II at 60–64y (nmol/L)	84.4 (53.3)	83.2 (57.9)	84.4 (50.3)	84.3 (51.7)	86.3 (55.3)
IGFBP-3 at 60–64y (nmol/L)*	120.2 ± 30.1	120.1 ± 30.1	121.5 ± 30.8	117.8 ± 28.6	121.5 ± 30.8
IGF-I/IGFBP-3 molar ratio at 60–64y	0.188 (0.080)	0.195 (0.085)	0.190 (0.082)	0.189 (0.075)	0.172 (0.074)
Δ IGF-I (%)	78.7 (37.0)	80.4 (36.2)	79.0 (37.2)	79.4 (43.6)	75.3 (36.8)
Δ IGF-II (%)	75.6 (51.4)	74.9 (54.1)	76.0 (46.7)	74.8 (54.7)	77.3 (49.3)
Δ IGFBP-3 (%)	60.4 (22.8)	59.3 (24.8)	59.9 (22.1)	61.4 (23.8)	61.3 (21.9)
Δ IGF-I/IGFBP-3 molar ratio	1.15 (0.65)	1.18 (0.66)	1.16 (0.59)	1.20 (0.71)	1.10 (0.60)
HbA1c (mmol/mol)	39 (5)	39 (4)	39 (5)	39 (5)	39 (5)
Blood glucose (mg/dl)	5.5 (0.9)	5.5 (0.75)	5.6 (0.9)	5.6 (0.9)	5.5 (1.0)
HDL (mmol/L)	1.54 (0.54)	1.52 (0.57)	1.55 (0.50)	1.55 (0.49)	1.54 (0.58)
LDL (mmol/L)	3.53 (1.35)	3.54 (1.25)	3.55 (1.41)	3.58 (1.25)	3.48 (1.41)
Total cholesterol (mmol/L)	5.65 (1.47)	5.53 (1.38)	5.70 (1.49)	5.74 (1.53)	5.66 (1.56)
Triglycerides (mmol/L)	1.08 (0.77)	1.05 (0.73)	1.12 (0.81)	1.12 (0.69)	1.07 (0.75)
Blood potassium (mmol/L)	4.23 (0.36)	4.26 (0.34)	4.24 (0.36)	4.22 (0.36)	4.20 (0.39)
Blood sodium (mmol/L)	140.4 (2.7)	140.2 (2.5)	140.4 (2.7)	140.4 (2.7)	140.4 (2.8)
**Clinical**					
Diabetes, *n* (%)	317 (21.9)	71 (18.9)	83 (22.8)	87 (24.5)	76 (21.5)
Hypertension, *n* (%)	720 (49.7)	174 (46.3)	183 (50.3)	173 (48.7)	190 (53.8)
Heart disease, *n* (%)	273 (18.9)	63 (16.8)	60 (16.5)	71 (20.0)	79 (22.4)
Hyperthyroidism, *n* (%)	28 (1.9)	10 (2.7)	5 (1.4)	8 (2.3)	5 (1.4)
Hypothyroidism, *n* (%)	146 (10.1)	37 (9.8)	36 (9.9)	39 (11.0)	34 (9.6)
**Smoking**					
Ex-smoker, *n* (%)	746 (51.5)	191 (50.8)	201 (55.2)	179 (50.4)	175 (49.6)
Current smoker, *n* (%)	126 (8.7)	34 (9.0)	24 (6.6)	36 (10.1)	32 (9.1)
Alcohol intake (g)	26.1 (23.7)	27.4 (22.6)	25.2 (23.7)	26.1 (27.9)	25.6 (22.5)
Physical activity (4 weeks), *n* (%)^b^	588 (40.6)	148 (39.4)	139 (38.2)	155 (43.7)	146 (41.4)
Physical activity (12 months), *n* (%)^b^	1,273 (87.9)	337 (89.6)	323 (88.7)	308 (92.4)	305 (86.4)
**Medications**					
Antibiotics, *n* (%)	31 (2.1)	6 (1.6)	11 (3.0)	8 (2.3)	6 (1.7)
Antidepressants, *n* (%)	104 (7.2)	28 (7.4)	24 (6.6)	24 (6.8)	28 (7.9)
Antihypertensives, *n* (%)	354 (24.4)	67 (17.8)	90 (24.7)	87 (24.5)	110 (31.2)
Antipsychotics, *n* (%)	9 (0.6)	1 (0.3)	3 (0.8)	1 (0.3)	4 (1.1)

a * Defined as manual for socioeconomic position (SEP) classes IIIM-V and non-manual for SEP classes I-IIINM*.

b *Physical activity represents the self-reporting on one form of physical activity undertaken at least once within the previous 4 weeks and 12 months*.

*All participants who had both ECG and IGF-I blood tests are presented*.

Results are reported as counts (%) for categorical variables, mean ± 1 standard deviation for normally distributed variables (*) or median (interquartile range) for non-normal variables. BMI, body mass index; DBP, diastolic blood pressure; ECG, electrocardiography; EF, ejection fraction; HbA1c, glycated hemoglobin; HDL, high density lipoprotein; IGF-I, insulin-like growth factor-I; IGF-II, insulin-like growth factor-II; IGFBP-3, insulin-like growth factor binding protein 3; LDL, low density lipoprotein; LV, left ventricular; MAP, mean arterial pressure; SBP, systolic blood pressure; SEP, socio-economic position; QTc, corrected QT interval using Hodges' formula.

### IGFs and IGFBP-3 at 53y in Relation to QTc Interval at 60–64y

On univariate analysis (Table 2) none of the IGF variables at 53y was significantly associated with a prolonged QTc interval a decade later (60–64y).

**Table 2:** Univariate associations between QTc and clinicodemographic data at age 60–64.

	**QTc (ms)**	
**Variables**	**β-coefficient (95% CI)**	***p*-value**
Age	−0.14 (−1.09, 0.80)	0.767
IGF-I at 63y (nmol/L)	−0.30 (−0.44, −0.17)	**<0.001**
IGF-II at 63y (nmol/L)	– 0.007 (−0.03, 0.02)	0.615
IGFBP-3 at 63y (nmol/L)	– 0.004 (−0.04, 0.03)	0.832
IGF-I/IGFBP-3 molar ratio at 60–64y	−28.9 (−41.93, −15.50)	**<0.001**
IGF-I at 53y (nmol/L)	−0.05 (−0.18, 0.08)	0.426
IGF-II at 53y (nmol/L)	0.02 (−0.02, 0.05)	0.349
IGFBP-3 at 53y (nmol/L)	−0.01 (−0.04, 0.02)	0.379
IGF-I/IGFBP-3 molar ratio at 53y	1.87 (−15.17, 19.15)	0.832
Δ IGF-I	−0.04 (−0.08, −0.008)	**0.019**
Δ IGF-II	−0.01 (−0.03, 0.01)	0.404
Δ IGFBP-3	0.01 (−0.04, 0.06)	0.734
Δ IGF-I/IGFBP-3 molar ratio	−2.44 (−4.17,−0.67)	**0.007**
HbA1c (mmol/mol)	0.05 (−0.10, 0.21)	0.508
Blood glucose (mg/dl)	0.18 (−0.74, 1.11)	0.708
Sex (male)	−5.36 (−7.48,−3.23)	**<0.001**
BMI (kg/m^2^)	0.50 (0.26, 0.73)	**<0.001**
SEP (non-manual)	−2.55 (−4.86,−0.25)	**0.030**
Smoking:	-	-
Ex-smoker	0.15 (−2.00, 2.29)	0.894
Current smoker	−0.65 (−4.42, 3.16)	0.737
Heart rate (bpm)	−0.17 (−0.27,−0.07)	**0.001**
SBP (mmHg)	0.03 (−0.03, 0.09)	0.292
DBP (mmHg)	0.03 (−0.09, 0.14)	0.649
MAP (mmHg)	0.03 (−0.07, 0.12)	0.589
LV mass (g)	0.02 (0.006, 0.03)	**0.006**
LV EF (%)	−0.06 (−0.21, 0.09)	0.444
HDL (mmol/L)	0.50 (−2.15, 3.15)	0.715
LDL (mmol/L)	0.16 (−0.91, 1.22)	0.775
Total cholesterol (mmol/L)	0.40 (−0.50, 1.30)	0.384
Triglycerides (mmol/L)	0.26 (−1.23, 1.76)	0.734
Hyperthyroidism	−2.35 (−10.00, 5.48)	0.551
Hypothyroidism	0.55 (−3.01, 4.14)	0.762
Blood potassium (mmol/L)	−8.29 (−11.81, −4.78)	**<0.001**
Blood sodium (mmol/L)	0.20 (−0.24, 0.65)	0.379
Hypertension	2.55 (0.42, 4.69)	**0.019**
Diagnosed Heart disease	3.74 (1.00, 6.49)	**<0.001**
Alcohol intake (g)	−0.02 (−0.08, 0.04)	0.543
Physical activity (4 weeks)	−0.01 (−2.19, 2.17)	0.990
Physical activity (12 months)	−2.21 (−5.51, 1.08)	0.190
Antibiotics	0.30 (−1.93, 7.78)	0.938
Antidepressants	2.25 (−1.89, 6.44)	0.289
Antihypertensives	5.09 (2.60, 7.59)	**<0.001**
Antipsychotics	10.18 (−3.47, 24.42)	0.153

*Analyses are by generalized linear models. Significant p-values are highlighted in bold*.

*bpm, beats per minute; CI, confidence interval; β-coefficient, regression coefficient. BMI, body mass index; DBP, diastolic blood pressure; ECG, electrocardiography; EF, ejection fraction; HbA1c, glycated hemoglobin; HDL, high density lipoprotein; IGF-I, insulin-like growth factor-I; IGF-II, insulin-like growth factor-II; IGFBP-3, insulin-like growth factor binding protein 3; LDL, low density lipoprotein; LV, left ventricular; MAP, mean arterial pressure; SBP, systolic blood pressure; SEP, socio-economic position; QTc, corrected QT interval using Hodges' formula*.

### IGFs and IGFBP-3 at 60–64y in Relation to QTc Interval at 60–64y

On univariate analysis (Table 2) low IGF-I and low IGF-I/IGFBP-3 molar ratio showed an association with QTc prolongation at 60–64y (β −0.30 ms/nmol/L [−0.44, −0.17], *p* < 0.001 and β −28.9 [−41.93, −15.50], *p* < 0.001), but IGF-II and IGFBP-3 alone showed no association with QTc. After multivariable adjustment in imputed models, low IGF-I (β −0.21 ms/nmol/L [−0.39, −0.04] *p* = 0.017; representing a multiplicative increase in QTc duration of ≈0.81 ms (exp[-0.21]) per 1 ms/nmol/L decrease in serum IGF-I levels), and low IGF -I/IGFBP-3 molar ratio at 60–64y (β −20.14 ms/unit [−36.28, −3.99] *p* = 0.015) were independently associated with QTc (Table 3). Complete case analysis support these inferences (Table 4).

**Table 3:** Multivariable imputed model exploring the association between IGF-I at 60-64y, IGF-I/IGFBP-3 molar ratio at 60-64y, ΔIGF-I and ΔIGF-I/IGFBP-3 ratio with QTc outcomes (only fully adjusted results for Model 4 are shown here).

	**QTc (ms) at 60-64y**											
**Variable**		**β-coefficient (95% CI)**	***p*-Value**		**β-coefficient (95% CI)**	***p*-Value**		**β-coefficient (95% CI)**	***p*-value**		**β-coefficient (95% CI)**	**p-value**
Biomarker	***IGF-I 60–64y***	−0.21 (−0.39, −0.04)	**0.017**	***IGF-I/IGFBP-3*** ***ratio at 60-64y***	−20.14 (−36.28, −3.99)	**0.015**	***ΔIGF-I***	−0.05 (−0.10, −0.002)	**0.042**	***ΔIGF-I/*** ***IGFBP-3***	−2.15 (−4.23, −0.09)	**0.041**
Age		−0.31 (−1.53, 0.91)	0.620		−0.241 (−1.47, 0.99)	0.701		−0.51 (−1.83, 0.80)	0.444		−0.54 (−1.86, 0.77)	0.417
Sex (Male)		−9.65 (−12.65, −6.65)	**<0.001**		−9.29 (−12.35, −6.22)	**<0.001**		−10.82 (−13.88, −7.75)	**<0.001**		−10.63 (−13.72, −7.54)	**<0.001**
SEP		−1.37 (−4.33, 1.59)	0.364		−1.69 (4.65, 1.27)	0.262		−1.45 (−4.54, 1.65)	0.359		−1.64 (−4.75, 1.48)	0.302
BMI		−0.04 (−0.37, 0.29)	0.820		−0.02 (−0.35, 0.31)	0.910		0.06 (−0.30, 0.41)	0.757		0.09 (−0.27, 0.45)	0.626
K^+^		−5.70 (−10.23, −1.18)	**0.014**		−5.96 (−10.48, −1.43)	**0.010**		−4.90 (−9.61, −0.19)	**0.042**		−4.82 (−9.53, −0.10)	**0.045**
LV mass		0.04 (0.02, 0.06)	**<0.001**		0.04 (0.02, 0.06)	**<0.001**		0.04 (0.03, 0.06)	**<0.001**		0.04 (0.03, 0.06)	**<0.001**
Heart Disease		2.29 (−1.20, 5.77)	0.198		2.01 (−1.48, 5.50)	0.260		1.69 (−1.96, 5.33)	0.364		1.44 (−2.24, 5.12)	0.443
Hypertension		0.954 (−1.84, 3.75)	0.503		0.86 (−1.94, 3.66)	0.548		1.22 (−1.66, 4.10)	0.405		1.40 (−1.49, 4.28)	0.343

*Analyses are by generalized linear models after centering for age. Significant p-values are highlighted in bold*.

*bpm, beats per minute; CI, confidence interval; β-coefficient, regression coefficient. BMI, body mass index; DBP, diastolic blood pressure; ECG, electrocardiography; EF, ejection fraction; HbA1c, glycated hemoglobin; HDL, high density lipoprotein; IGF-I, insulin-like growth factor-I; IGF-II, insulin-like growth factor-II; IGFBP-3, insulin-like growth factor binding protein 3; LDL, low density lipoprotein; LV, left ventricular; MAP, mean arterial pressure; SBP, systolic blood pressure; SEP, socio-economic position; QTc, corrected QT interval using Hodges' formula*.

**Table 4:** Multivariable regression (complete case analysis) for IGF-I at 60-64y, IGF-I/BP3 molar ratio at 60-64y, ΔIGF-I, ΔIGF-I/BP3 and exposures of interest with QTc.

	**Model 1**		**Model 2**		**Model 3**		**Model 4**	
	**β-coefficient** **[95% CI]**	***p*-Value**	**β-coefficient** **[95% CI]**	***p*-Value**	**β-coefficient** **[95% CI]**	***p*-Value**	**β-coefficient** **[95% CI]**	***p*-Value**
***IGF-I 60-64y***	−0.27 (−0.42, −0.13)	**<0.001**	−0.27 (−0.88, 0.99)	**<0.001**	−0.25 (−0.39, −0.11)	**<0.001**	– 0.23 (– 0.40, – 0.06)	**0.008**
Age	0.02 (−0.92, 0.95)	0.975	0.05 (−0.41, −0.12)	0.909	−0.13 (−1.07, 0.79)	0.776	– 0.36 (– 1.56, 0.83)	0.553
Sex (male)	−5.00 (−7.22, −2.79)	**<0.001**	−5.04 (−7.25, −2.82)	**<0.001**	−4.62 (−6.84, −2.41)	**<0.001**	– 9.10 (– 12.00, – 6.20)	**<0.001**
SEP (manual work)			−2.73 (−5.12, −0.34)	**0.025**	−2.31 (−4.69, 0.06)	0.057	– 1.56 (– 4.34, 1.21)	0.271
BMI					0.45 (0.20, 0.69)	**<0.001**	– 0.007 (– 0.33, 0.32)	0.969
K^+^					−6.93 (−10.57, −3.29)	**<0.001**	– 5.43 (– 9.81, – 1.05)	**0.016**
LV mass							0.04 (0.02, 0.06)	**<0.001**
Heart disease							3.27 (– 0.04, 6.60)	0.054
Hypertension							1.18 (– 1.55, 3.91)	0.398
***IGF-I/IGF-BP3 ratio 60-64y***	−24.31 (−38.28, −9.85)	**0.001**	−24.82 (−38.81, −10.35)	**<0.001**	−24.97 (−38.87, −10.60)	**<0.001**	−20.32 (−35.60, −4.36)	**0.012**
Age	0.10 (−0.86, 1.05)	0.842	0.15 (– 0.80, 1.10)	0.756	−0.03 (−0.98, 0.91)	0.943	−0.31 (−1.51, 0.89)	0.616
Sex (male)	−4.73 (−7.00, −2.46)	**<0.001**	−4.73 (−7.00, −2.47)	**<0.001**	−4.26 (−6.52, −1.99)	**<0.001**	−8.74 (−11.71, −5.77)	**<0.001**
SEP (manual work)			−3.03 (−5.42, −0.64)	**0.013**	−2.60 (−4.98, −0.23)	**0.032**	−1.88 (−4.66, 0.89)	0.183
BMI					0.46 (0.22, 0.71)	**<0.001**	0.02 (−0.31, 0.34)	0.914
K^+^					−7.27 (−10.91, −3.63)	**<0.001**	−5.64 (−10.03, −1.26)	**0.012**
LV mass							0.04 (0.02, 0.06)	**<0.001**
Heart disease							3.05 (−0.27, 6.39)	0.073
Hypertension							1.08 (−1.65, 3.82)	0.438
***Δ** **IGF-I***	−0.04 (−0.08, −0.002)	**0.039**	−0.04 (−0.08, −0.003)	**0.034**	−0.04 (−0.08, −0.002)	**0.039**	−0.05 (−0.09, −0.003)	**0.038**
Age	−0.268 (−1.32, 0.78)	0.617	−0.19 (−1.24, 0.86)	0.719	−0.41 (−1.46, 0.63)	0.439	−0.73 (−2.04, 0.57)	0.270
Sex (male)	−6.02 (−8.34, −3.70)	**<0.001**	−6.04 (−8.36, −3.73)	**<0.001**	−5.67 (−7.98, −3.36)	**<0.001**	−10.20 (−13.21, 7.20)	**<0.001**
SEP (manual work)			−2.51 (−5.07, 0.04)	0.054	−2.16 (−4.70, 0.37)	0.094	−1.29 (−4.23, 1.65)	0.390
BMI					0.55 (0.29, 0.81)	**<0.001**	0.12 (−0.24, 0.47)	0.519
K^+^					−6.62 (−10.49, −2.75)	**<0.001**	−4.49 (−9.08, 0.09)	0.057
LV mass							0.05 (0.03, 0.06)	**<0.001**
Heart disease							2.80 (−0.71, 6.33)	0.120
Hypertension							1.56 (−1.28, 4.40)	0.283
***Δ** **IGF-I/IGF-BP3 ratio***	−1.90 (−3.72, −0.03)	**0.046**	−2.02 (−3.84, −0.14)	**0.034**	−2.26 (−4.06, 0.40)	**0.017**	−2.14 (−4.13, −0.06)	**0.042**
Age	−0.37 (−1.42, 0.66)	0.481	−0.285 (−1.33, 0.76)	0.593	−0.44 (−1.48, 0.59)	0.399	−0.78 (−2.08, 0.52)	0.240
Sex (male)	−5.98 (−8.32, −3.64)	**<0.001**	−6.00 (−8.34, −3.66)	**<0.001**	−5.61 (−7.94, −3.28)	**<0.001**	−10.02 (−13.05, 7.00)	**<0.001**
SEP (manual work)			−2.57 (−5.15, 0.005)	0.051	−2.22 (−4.78, 0.33)	0.089	−1.45 (−4.41, 1.50)	0.336
BMI					0.58 (0.31, 0.84)	**<0.001**	0.15 (−0.21, 0.50)	0.415
K^+^					−6.55 (−10.44, −2.66)	**0.001**	−4.40 (−8.99, 0.195)	0.063
LV mass							0.04 (0.03, 0.06)	**<0.001**
Heart disease							2.61 (−0.92, 6.17)	0.149
Hypertension							1.72 (−1.12, 4.57)	0.236

*Analyses are by generalized linear models after centering for age. Significant p-values are highlighted in bold*.

*Heart rate was not included in the multivariable model as it was already accounted for by the QTc correction using Hodges' formula. Four models were used from left to right in a stepwise manner. Model 1 was centered for age and adjusted for sex, Model 2 was adjusted as for model 1 + SEP, Model 3 was adjusted as for model 2 + clinical covariates (BMI and K^+^) and Model 4 was adjusted as for Model 3 + cardiac covariates (LV mass, heart disease and hypertension)*.

*bpm, beats per minute; CI, confidence interval; β-coefficient, regression coefficient. BMI, body mass index; DBP, diastolic blood pressure; ECG, electrocardiography; EF, ejection fraction; HbA1c, glycated hemoglobin; HDL, high density lipoprotein; IGF-I, insulin-like growth factor-I; IGF-II, insulin-like growth factor-II; IGFBP-3, insulin-like growth factor binding protein 3; LDL, low density lipoprotein; LV, left ventricular; MAP, mean arterial pressure; SBP, systolic blood pressure; SEP, socio-economic position; QTc, corrected QT interval using Hodges' formula*.

Based on these multiple imputation models, independent associations with QTc were also confirmed for other previously known covariates: female sex (β 9.65 [6.65, 12.65] *p* < 0.001), increased left ventricular mass (β 0.04 ms/g [0.02, 0.06] *p* < 0.001) and blood potassium levels (β −5.70 ms/mmol/L [−10.23, −1.18] *p* = 0.014).

### ΔIGFs and ΔIGFBP-3 in Relation to QTc Interval Over a Decade

**Figure 2:**
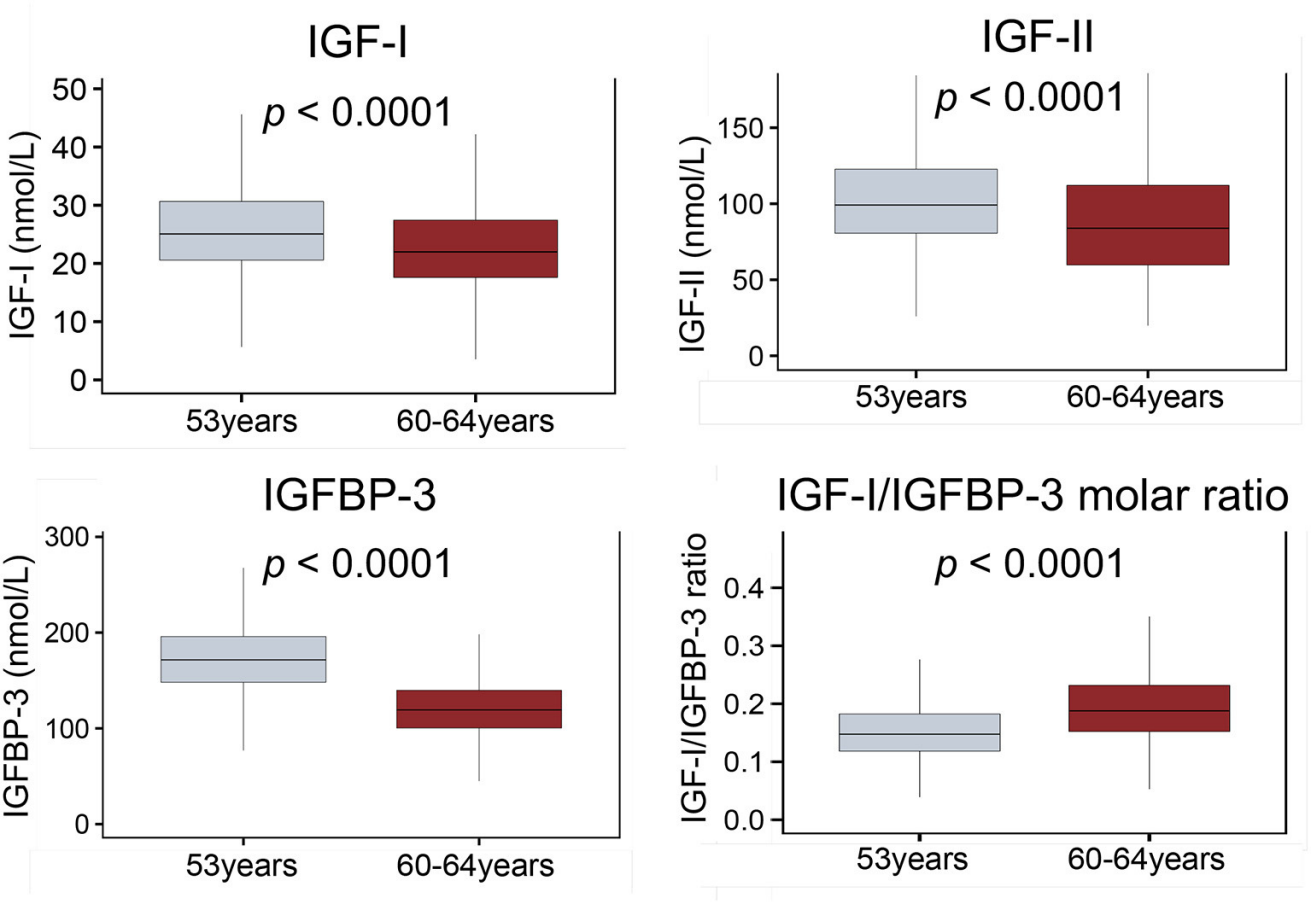
Boxplots comparing circulating levels of IGF-I, IGF-II, IGFBP-3 and IGF-I/IGFBP-3 molar ratio at 53 and 60–64 years. Whiskers indicate variability outside the third and first quartiles [75th and 25th percentiles] represented as hinges around the median [bold midline]. *p*-Values derived from Mann-Whitney tests. ECG, electrocardiography; IGF-I, insulin-like growth factor 1; IGF-BP3, insulin-like growth factor binding protein 3.

IGF-I, IGF-II and IGFBP-3 levels decreased with age (all *p* < 0.001; Figure 2) while the IGF-I/IGFBP-3 ratio increased with age (*p* < 0.001; Figure 2).

On univariate analysis (Table 2) a steeper decline in IGF-I, ΔIGF-I, (β −0.04 ms/nmol/L/10 years [−0.08, −0.008], *p* = 0.019) and a shallower rise in ΔIGF-I/IGFBP-3 (β −2.44 ms/unit/10 years [−4.17, −0.67], *p* = 0.007) were associated with a longer QTc. These associations persisted in complete case analysis multivariable models (β −0.05 ms/nmol/L/10 years [−0.09, −0.003], *p* = 0.038 and β −2.14 ms/unit/10 years [−4.13, −0.06], *p* = 0.042) and in fully adjusted imputed models (β −0.05 ms/nmol/L/10 years [−0.10, −0.002], *p* = 0.042 and β −2.16 ms/unit/10 years [−4.23, −0.09], *p* = 0.041). As before, the multiple imputation analyses remained significant for sex, left ventricular mass and blood potassium levels.

### Sensitivity Analyses

In imputed models, observed associations between IGF-I/IGFBP-3 molar ratio at 60–64y and ΔIGF-I/IGFBP-3 ratio with QTc, were only slightly attenuated after removing the 273 participants with known cardiovascular disease (respectively: β −17.60 ms/unit [−34.41, −0.80], *p* = 0.040 and β −2.35 ms/unit/10 years [−4.44, −0.24], *p* = 0.029, Supplementary Table 3).

## Discussion

In a UK-based sample of older persons aged 60–64 years, declining serum levels and bioavailability of IGF-I (previously known as Somatomedin C) associated with QTcprolongation, a well-established risk factor for sudden death, independent of sex, SEP, BMI, LV mass, heart disease and hypertension.

In several studies it has been shown that reduced levels of IGF-I were associated with increased risk of cardiovascular disease (44, 45). IGF-I is thought to protect cardiac myocytes from arrhythmogenesis and apoptosis by activating the PI3-K/Akt cell survival intracellular signaling (21), although the exact mechanism remains to be fully elucidated, with a possibility that channel transcription might be affected. The PI3-K pathway activates the serine/threonine protein kinase Akt, and Protein Kinase C, promoting cardiovascular homeostasis, neuroprotection, survival, gene expression, and insulin activity (19, 46). The rapid delayed rectifier potassium channels that influence cardiac repolarization in cardiomyocytes are regulated by PI3-K (21, 22, 47). These channels (I_Ks_, I_Kr_ and the atrial specific I_Kur_) conduct outward potassium currents during the plateau phase of the action potential (48). Mutations in the genes encoding delayed rectifiers disrupt normal cardiac repolarization and lead to various cardiac rhythm disorders, including congenital long QT syndrome. Late sodium currents, I_NaL_, were shown to be activated by another a downward pathway of PI3-K, the serum- and glucocorticoid-regulated kinase (SGK), which phosphorylates the sodium channels on the cardiomyocyte surface. GSK was shown to phosphorylates neural precursor cell expressed developmentally down-regulated protein 4 (NEED4), blocking the ubiquitination of the sodium channel (49, 50). These two channels, I_Kr_ and I_Na_, were thought to finely interact to maintain a correct ventricular repolarization (51). Reduced IGF-I binding to its receptors on cardiomyocytes, has been shown to decrease the activation of the PI3-K/Akt pathway in animal models (22) thus prolonging the action potential duration and also the QTc. One factor that is thought to mediate the electrophysiological effect of IGF-I is nuclear factor erythroid 2-related factor 2 (Nrf2), which normally induces the transcription of cytoprotective enzymes involved in antioxidative pathways (52, 53) that have the ability to suppress ventricular arrhythmias. Evidence suggests that IGF-I regulates the levels of Nrf2 expression and modulates its transcriptional activity via the PI3K/Akt pathway (54). Based on the results from our current study, it is plausible therefore, that in older persons, declining levels and bioavailability of IGF-I reduce expression of Nrf2, prolonging QTc, and increasing the propensity for malignant arrhythmias.

IGF-I and IGF-II are mainly produced by the liver under the influence of growth hormone and nutrition (19, 55, 56). They have a similar structure to insulin with a similar direct effect on the body's glucose metabolism (57, 58), but are found in much higher concentrations in the blood where they are bound to binding proteins (mainly to isoform IGFBP-3), also produced by the liver. Binding to IGFBP-3 increases its half-life and modulates receptor binding. IGF-I fulfills several important functions in the human body by reaching several targets, where both insulin and IGF-1 receptors are found. An imbalance in serum IGF-I levels has been associated with a variety of negative effects, in several body systems, including obesity, diabetes and atherosclerosis. Reduced levels of IGF-I increase risk of hypertension, inflammation and endothelial dysfunction as normal IGF-I levels were shown to be protective, stimulating the release of nitric oxide, a vasodilator, and promoting cell proliferation and differentiation (46, 59). Quantitatively, IGF-II is the predominant circulating IGF, present in adults at a concentration up to three times that of IGF-I (as noted in our cohort). In mammals, IGF-I mediates the growth promoting effects of growth hormone during postnatal life and throughout adulthood. It influences cardiomyocytes and the cardiac action potential as described above, but IGF-II is more involved in placental and fetal growth (including cardiac development) in utero (60), is less growth hormone dependent than IGF-I (61), and is not known to influence the cardiac action potential in mature cardiomyocytes. Indeed, we found no statistically significant association between circulating levels of IGF-II and QTc interval at either time point. As IGF-I fulfills important somatic growth function it reaches its highest levels during teenage years, with levels subsequently decreasing with age (16, 17) in a highly variable and individual process that is related to fat mass (16), sex, diet and hormonal status. Among its various functions, IGF-I protects against inflammation, hypertension, endothelial and β-cell dysfunction (46, 59, 62), inhibits growth hormone hypersecretion and suppresses insulin secretion whilst enhancing insulin's action (63). An imbalance in serum IGF-I levels has been associated with obesity, diabetes and atherosclerosis (46, 59).

The six IGF-binding proteins (IGFBP-1-6) have both IGF-dependent and independent functions (64, 65). IGFBP-3 is the most abundant of the six IGFBPs in the circulation. Low IGFBP-3 levels have been associated with adverse cardiovascular effects, increased vascular disease and higher risk of coronary events (66). IGFBPs serve not only to transport IGFs in the circulation but also to prolong their half-lives, modulate their tissue specificity, and to either potentiate or neutralize their biological actions at tissue level (65). Measuring free (unbound) IGF-I remains a challenge (52) and total measured serum IGF-I is not tantamount to bioavailable IGF-I (33): almost all circulating IGF-I is bound to IGFBPs leaving <1% of IGF-I in a free form bioavailable for receptor-binding (47). The molar ratio of total IGF-I to IGFBP-3 is widely used as the proxy for bioavailable IGF-I (67).

Our study examined whether the differential decline in IGFs, IGFBP-3 or IGF-I/IGFBP-3 ratio over a decade (53 to 60–64 years) put some older persons at higher risk of QTc prolongation. Results showed that adults who experienced a steeper decline in IGF-I over a decade of later life were at higher risk of QTc prolongation. With age, levels of IGFBP-3 decline more steeply than IGF-I which is why the IGF-I/IGFBP-3 ratio (and therefore IGF-I bioavailability) appears to increase (32) (Figure 2). We show that older persons whose molar ratios increased least over a decade—implying less bioavailable IGF-I overall—had prolonged QTc compared to those who had higher IGF-I bioavailability. This aligns with other recently published data showing how low levels of IGFBP-3 increased the risk of cardiovascular disease and mortality (18) and how low IGF-I/IGFBP-3 ratios increased the risk of metabolic syndrome and insulin resistance (68). The fact that after removing persons with cardiovascular disease in the sensitivity analysis, only the 60–64y IGF-I/IGFBP-3 molar ratio and ΔIGF-I/IGFBP-3 ratio retained significant association with QTc, adds credence to the notion that it is the free, bioavailable IGF-I which most strongly determines the electrophysiological effect observed. Therefore, the IGF-I/IGFBP-3 ratio has to maintained at safe levels to avoid adverse metabolic, cardiovascular and neoplastic effects (69).

Our finding, that low levels of IGF-I relate to QTc prolongation and therefore higher sudden cardiac death risk, fits with several other known adverse cardiovascular effects of IGF-I deficiency, that include accelerated cardiovascular aging, reduced cardiac contractility, hypertrophy, hypertension, coronary disease and even atrial fibrillation (19, 53, 70). Conversely, normal IGF-I levels appear to be cardioprotective, by stimulating the release of vasodilatory nitric oxide, and by promoting cell proliferation and differentiation (46, 59). In mice IGF-I receptor deficiency has been associated with cardiomyopathy and heart failure (53).

Previous works that failed to account for confounders, have observed an association between diabetes or insulin resistance with QTc prolongation (71–75), but we found no association between fasting blood glucose levels or HbA1c with QTc at age 60-64. The mechanism of QTc prolongation in insulin resistance is still unclear but it is known that insulin may increase the transmembrane potential of cardiomyocytes by activating the electrogenic Na^+^/K^+^-ATPase leading to hyperpolarization and therefore QT prolongation (76).

We found an association between low blood potassium levels and QTc prolongation, as anticipated. Important cardiomyocyte-specific mechanisms are the main determinants of hypokalemia-induced QT prolongation (77) in addition to altering autonomic nervous system activity (78, 79). Potassium deficiency, as shown experimentally in a legacy study on rats (80), also reduces IGF-I, so it potentially confounds the relationship between IGF-I and QTc. Several previous studies (81–83) explored an association between increased LV mass and prolonged QTc, supporting our evidence, that LV mass is also an important determinant of QTc.

### Limitations

As with most epidemiological studies, the main limitation is unmeasured or residual confounding as this precludes causal inferences. The extent of missing data in our study (Supplementary Tables 1, 2) was small and we go on to show that key associations between biomarkers and QTc persisted after multiple imputation. Multiple imputation, however, cannot account for sample selection. The inclusion of British people born during the same week in 1946, leads to issues with external validity as the findings may not be applicable to non-British populations.

Earlier measurements of IGFs and IGFBP-3 (pre-53y), as well as measurements at shorter time intervals could have helped detect temporal trends better in this cohort. Levels of other IGFBPs that may have more direct effects on cardiac function were not measured (20, 84). The broader metabolic effects of IGF-I linked to other metabolic markers such as leptin, insulin or glucose, could not be explored over the decade as measurements of these additional blood markers were not available at the age of 53y. Several methods for QT correction exist and although the Bazett's formula remains one of the most widely used methods, it is known to overcorrect the QT interval (85, 86). Other correction methods (86, 87), including Hodges' formula used here, have been shown to be better (86). Comparisons between the various QTc correction methods was beyond the scope of this study, yet we go on to show in the sensitivity analysis (Supplementary Table 4) that key biomarker associations with QTc persisted after additional adjustment for heart rate in the multivariable models.

## Conclusion

In a large older-age population-based cohort, declining levels and bioavailability of IGF-I associate with prolonged QTc interval. As QTc prolongation is known to be associated with increased risk for sudden death even in apparently healthy people, further work is needed to understand and preserve the potentially anti-arrhythmic effects of IGF-I in older age.

## Data Availability Statement

NSHD data is available through the Medical Research Council Skylark website (https://skylark.ucl.ac.uk/Skylark) and full details on the archived data is available at: https://www.nshd.mrc.ac.uk/data.

## Ethics Statement

The studies involving human participants were reviewed and approved by Greater Manchester Local Research Ethics Committee and the Scotland Research Ethics Committee. The patients participants provided their written informed consent to participate in this study.

## Author Contributions

CC and GC conceived of the study. CC wrote the manuscript and analyzed the data. JM, JH, NC, and AH provided expert review of the manuscript. All authors contributed to the article and approved the submitted version.

## Funding

GC and JM are supported by the Barts Charity HeartOME grant (MGU0427). GC was supported by British Heart Foundation (MyoFit46 Special Programme Grant SP/20/2/34841) and by the NIHR UCL Hospitals Biomedical Research Center. The NSHD cohort was funded by the UK MRC (program codes MC_UU_12019/1; MC_UU_12019/4; MC_UU_12019/5). JM is directly and indirectly supported by the UCL Hospitals NIHR BRC and Biomedical Research Unit at Barts Hospital, respectively. AH receives support from the British Heart Foundation, the Economic and Social Research Council (ESRC), the Horizon 2020 Framework Programme of the European Union, the National Institute on Aging, the National Institute for Health Research University College London Hospitals Biomedical Research Center and the UK MRC.

## Conflict of Interest

The authors declare that the research was conducted in the absence of any commercial or financial relationships that could be construed as a potential conflict of interest.

## Publisher's Note

All claims expressed in this article are solely those of the authors and do not necessarily represent those of their affiliated organizations, or those of the publisher, the editors and the reviewers. Any product that may be evaluated in this article, or claim that may be made by its manufacturer, is not guaranteed or endorsed by the publisher.
