# Supplementary material for: Declining Levels and Bioavailability of IGF-I in Cardiovascular Aging Associate With QT Prolongation–Results From the 1946 British Birth Cohort
Source: Front Cardiovasc Med. 2022 Apr 22;9:863988. doi: 10.3389/fcvm.2022.863988 (PMC9072634; doi:10.3389/fcvm.2022.863988)
Supplement: Supplementary file 1 [file Data_Sheet_1.pdf]

SUPPLEMENTARY MATERIAL FOR:

Declining levels and bioavailability of IGF-I in cardiovascular aging associate with QT prolongation–results from the 1946 British Birth Cohort

Christos Charalambous[1], James C Moon[2,3], Jeffrey MP Holly[4,5], Nishi Chaturvedi[1], Alun D Hughes[1,2], Gabriella Captur*[1,2,6]

Author Affiliations:

[1]UCL MRC Unit for Lifelong Health and Ageing, University College London, Fitzrovia, London WC1E 7HB

[2]UCL Institute of Cardiovascular Science, University College London, Gower Street, London WC1E 6BT, UK

[3]Cardiac MRI Unit, Barts Heart Centre, West Smithfield, London, EC1A 7BE

[4] National Institute for Health Research (NIHR) Bristol Nutrition Biomedical Research Unit, Level 3, University Hospitals Bristol Education & Research Centre, Upper Maudlin Street, Bristol, BS2 8AE, UK

[5] School of Translational Health Sciences, Bristol Medical School, Faculty of Health Sciences, Southmead Hospital, University of Bristol, Bristol, BS10 5NB, UK

[6]The Royal Free Hospital, Centre for Inherited Heart Muscle Conditions, Cardiology Department, Pond Street, Hampstead, London NW3 2QG, UK

Online Table S1. Missing values per metabolic covariate in addition to the complete exposure-outcome pair.

**Metabolic marker – Outcome pair**	**QTc**[a] n (%)
**IGF-I at 60-64y**	0 (0.00)
**IGF-II at 60-64y**	0 (0.00)
**IGF-BP3 at 60-64y**	2 (0.14)
**IGF-I/IGF-BP3 molar ratio at 60-64y**	2 (0.14)
**IGF-I at 53y**	187 (12.91)
**IGF-II at 53y**	187 (12.91)
**IGF-BP3 at 53y**	189 (13.05)
**IGF-I/IGFBP-3 molar ratio at 53y**	197 (13.60)
**Δ IGF-I**	187 (12.91)
**Δ IGF-II**	187 (12.91)
**Δ IGFBP-3**	191 (13.19)
**Δ IGF-I/IGFBP-3**	199 (13.74)
**Glucose**	38 (2.62)
**HbA1c**	62 (4.28)

[a] Missing values for the covariates in addition to the complete exposure-outcome pairs.
Results are reported as counts.
HbA1c, glycated hemoglobin; IGFBP-3, insulin-like growth factor binding protein 3; IGF-I, insulin-like growth factor-I; IGF-II, insulin-like growth factor-II; QTc, corrected QT interval using Bazett's formula; y, years.

Online Table S2. Missing values per clinicodemographic covariate in addition to the complete exposure-outcome pair.

**EXPOSURE - QTc pair**	**IGF-I**[a] n (%)
**Age**	106 (7.32)
**Sex**	0 (0.00)
**BMI**	0 (0.00)
**SEP**	80 (5.52)
**LV mass$_i$**	395 (27.28)
**Potassium**	6 (0.41)
**Hypertension**	0 (0.00)
**Heart disease**	0 (0.00)

[a] Missing values for the covariates in addition to the complete exposure-outcome pair.
Results are reported as counts (%).
BMI, body mass index; LV, left ventricular; SEP, socioeconomic position. Other abbreviations as in **Online Table S1**.

Online Table S3. Imputed multivariable models for metabolic markers showing significant association with QTc at univariate analysis, after removing participants with known cardiovascular disease.

		**Multivariable Model**[a]	
**OUTCOME**	***n***	**Exponentiated *β* (95% CI)**	***p*-value**
**IGF-I**	1175	-0.15 (-0.35, 0.04)	0.112
**IGF-I/IGFBP-3 molar ratio at 60-64**	1175	-17.60 (-34.41, -0.80)	**0.040**
**Δ IGF-I**	1175	-0.04 (-0.09, 0.007)	0.093
**Δ IGF-I/IGFBP-3**	1175	-2.35 (-4.44, -0.24)	**0.029**

[a]The multiple imputation multivariable model was adjusted for age, sex, socio-economic position, body mass index, blood potassium, left ventricular mass and for the presence of hypertension.
All reported analyses here consisted of generalized linear models with gamma distribution and log link. Significant *p*-values are highlighted in bold.
β-coefficient, regression coefficient; CI, confidence interval. Other abbreviations as in **Online Table S1 and S2**.

Online Table S4. Imputed multivariable models for metabolic markers showing significant association with QTc and including heart rate as a covariate.

		Multivariable Model[a]	
**OUTCOME**	***n***	**Exponentiated *β* (95% CI)**	***p*-value**
**IGF-I**	1448	-0.20 (-0.38, -0.03)	**0.021**
**IGF-I/IGFBP-3 molar ratio at 60-64**	1448	-19.60 (-35.11, -3.01)	**0.020**
**Δ IGF-I/IGFBP-3**	1448	-1.97 (-3.81, -0.12)	**0.037**

[a]The multiple imputation multivariable models (Model 3 ΔIGF-I/IGFBP-3 and Model 4 for the rest) were adjusted for heart rate and for age, sex, socio-economic position, body mass index, blood potassium, left ventricular mass and presence of hypertension as appropriate.
All reported analyses here consisted of generalized linear models with gamma distribution and log link. Significant *p*-values are highlighted in bold.
*A*bbreviations as in **Online Table S1, S2 and S3.**
